# Enhancement of COPD biological networks using a web-based collaboration interface

**DOI:** 10.12688/f1000research.5984.2

**Published:** 2015-05-20

**Authors:** The sbv IMPROVER project team (in alphabetical order), Stephanie Boue, Brett Fields, Julia Hoeng, Jennifer Park, Manuel C. Peitsch, Walter K. Schlage, Marja Talikka, The Challenge Best Performers (in alphabetical order), Ilona Binenbaum, Vladimir Bondarenko, Oleg V. Bulgakov, Vera Cherkasova, Norberto Diaz-Diaz, Larisa Fedorova, Svetlana Guryanova, Julia Guzova, Galina Igorevna Koroleva, Elena Kozhemyakina, Rahul Kumar, Noa Lavid, Qingxian Lu, Swapna Menon, Yael Ouliel, Samantha C. Peterson, Alexander Prokhorov, Edward Sanders, Sarah Schrier, Golan Schwaitzer Neta, Irina Shvydchenko, Aravind Tallam, Gema Villa-Fombuena, John Wu, Ilya Yudkevich, Mariya Zelikman

**Affiliations:** 1 Philip Morris International R&D, Philip Morris Products S.A., Quai Jeanrenaud 5, 2000 Neuchâtel, Switzerland; 2 Selventa, One Alewife Center, Cambridge, MA, 02140, USA; 3 Systems Bioengineering Group - National Technical University of Athens, Ethniko Metsovio Politechnio, , 28is Oktovriou 42, Athina, 106 82, Greece; 4 Touro University Nevada, 874 American Pacific Drive, Henderson, NV, 89052, USA; 5 University of Pittsburgh, 4200 Fifth Ave, Pittsburgh, PA, 15260, USA; 6 Intelligent Data Analysis Group (DATAi), School of Engineering, Pablo de Olavide University, Ctra. de Utrera, km. 1 41013, Sevilla, Spain; 7 University of Toledo, 2801 W Bancroft St, Toledo, OH, 43606, USA; 8 Shemyakin & Ovchinnikov Institute of Bioorganic Chemistry, 16/10, Miklukho-Maklay str., Moscow, 117997, Russian Federation; 9 Private, Washington DC, USA; 10 USAMRIID, Attn: MCMR-UIZ-R, 1425 Porter Street, Frederick, MD, 21702-5011, USA; 11 Private, Boston, MA, USA; 12 Institute of Microbial Technology, Chandigarh, 160036, India; 13 Technion - Israel Institute of Technology, Technion City, Haifa, 3200003, Israel; 14 Louisville University, 301 E. Muhammad Ali Blvd, Louisville, KY, 40202, USA; 15 AnalyzeDat Consulting Services, Ernakulam, India; 16 Northeastern University, 360 Huntington Ave, Boston, MA, 02115, USA; 17 Edward Sanders Scientific Consulting, Rue du Clos 33, 2034 Peseux, Switzerland; 18 Massachusetts Institute of Technology, 77 Massachusetts Ave, Cambridge, MA, 02139, USA; 19 Kuban State University of Physical Education, Sport and Tourism, 161, Budennogo Str., Krasnodar City, 350015, Russian Federation; 20 Luxembourg Centre for Systems Biomedicine, University of Luxembourg, 7, avenue des Hauts-Fourneaux, 4362 Esch sur Alzette, Luxembourg; 21 Pablo de Olavide University, Ctra. de Utrera, km. 1 41013, Sevilla, Spain; 22 Cal Biopharma, 710 Somerset Ln, Foster Cit, CA, 94404-3728, USA; 23 University of Manchester, Oxford Rd, Manchester, M13 9PL, UK; 24 University of Washington, 1959 NE Pacific Street, HSB T-466, Seattle, WA, USA

**Keywords:** COPD, Chronic Obstructive Pulmonary Disease, network model, signaling pathway, crowdsourcing, crowd verification, jamboree, online collaboration

## Abstract

The construction and application of biological network models is an approach that offers a holistic way to understand biological processes involved in disease. Chronic obstructive pulmonary disease (COPD) is a progressive inflammatory disease of the airways for which therapeutic options currently are limited after diagnosis, even in its earliest stage. COPD network models are important tools to better understand the biological components and processes underlying initial disease development. With the increasing amounts of literature that are now available, crowdsourcing approaches offer new forms of collaboration for researchers to review biological findings, which can be applied to the construction and verification of complex biological networks. We report the construction of 50 biological network models relevant to lung biology and early COPD using an integrative systems biology and collaborative crowd-verification approach. By combining traditional literature curation with a data-driven approach that predicts molecular activities from transcriptomics data, we constructed an initial COPD network model set based on a previously published non-diseased lung-relevant model set. The crowd was given the opportunity to enhance and refine the networks on a website ( https://bionet.sbvimprover.com/) and to add mechanistic detail, as well as critically review existing evidence and evidence added by other users, so as to enhance the accuracy of the biological representation of the processes captured in the networks. Finally, scientists and experts in the field discussed and refined the networks during an in-person jamboree meeting. Here, we describe examples of the changes made to three of these networks: *Neutrophil Signaling*, *Macrophage Signaling*, and *Th1-Th2 Signaling*. We describe an innovative approach to biological network construction that combines literature and data mining and a crowdsourcing approach to generate a comprehensive set of COPD-relevant models that can be used to help understand the mechanisms related to lung pathobiology. Registered users of the website can freely browse and download the networks.

## Introduction

Molecular networks, such as the KEGG (Kyoto Encyclopedia of Genes and Genomes) pathways ^1, 2^, aid in understanding the complex interplay of signaling pathways in disease. Biological network models (hereafter referred to as networks) depict the inter-relationships between multiple signaling pathways and how their perturbations may dysregulate biological processes, eventually leading to the disease.

**Figure 1.**
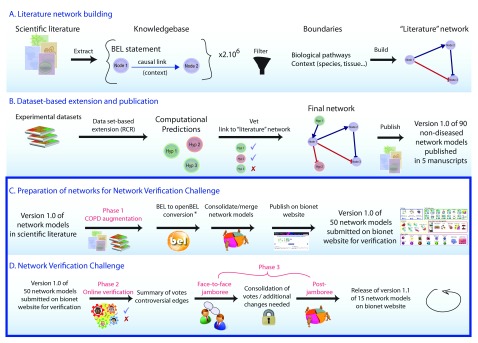
Network construction using a systems biology and crowdsourcing approach. Networks were constructed using published literature and data sets, and opened to the public for comment and editing in the Network Verification Challenge. The three phases of COPD network construction are shown. ( **A** and **B**) Phase 1: COPD augmentation using literature and data. ( **C** and **D**) Phase 2: Online verification by the public during an “open phase”, and Phase 3: Face-to-face jamboree meeting where scientists and subject matter experts gathered to discuss the networks and make final decisions for the next versions. * BEL was a proprietary language developed by Selventa. In the interest of the growing community of researchers using BEL, an openBEL language derived from BEL has been developed and released as open source. One of the main differences between the two is that in the openBEL, the namespace (i.e. databases in which the biological entity is defined) is clearly stated, allowing for a better standardization of used ontologies and databases.

**Figure 2.**
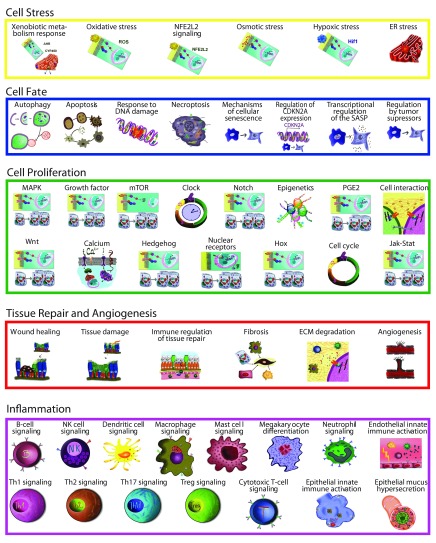
Fifty networks available during the network verification challenge and their associated biological processes.

In previously published reports, we described the construction of a set of 90 networks that captured a large range of biological processes relevant to non-diseased lung tissue ^3– 7^. The generation of this set of networks relied on both manual curation of published literature and a data-driven reverse causal reasoning (RCR) methodology ^8^ to augment the causal biological framework underlying the network architecture ( Figure 1). We used the Biological Expression Language (BEL) to represent precise biological relationships in a computable and standardized format ^8^. We have built upon this approach and describe here a unique, three-phase systems biology and crowdsourcing approach to construct a comprehensive set of 50 molecular networks that describe the biological processes relevant to chronic obstructive pulmonary disease (COPD) and lung biology ( Figure 2). COPD is the fourth leading cause of death worldwide and its incidence is increasing among chronic diseases in the USA ^9, 10^. COPD is a chronic, progressive inflammatory disease induced by cigarette smoking, inhalation of pollutants, dust, chemicals, or other foreign matter, which ultimately manifests as tissue destruction in the alveolar compartments and airflow limitation, leading to reduced oxygen exchange ^11– 15^. COPD affects a wide spectrum of biological processes in lung tissue, such as oxidative stress, inflammation, apoptosis, proliferation, and senescence ^16, 17^. Understanding the mechanisms involved in these processes is important in understanding the onset of the disease and in identifying drug targets to develop effective COPD treatments ^18, 19^. As recently reported by the Global Initiative for Chronic Obstructive Lung Disease (GOLD), current pharmacologic therapies cannot cure the disease but only reduce the symptoms, and the frequency and severity of exacerbations, i.e., slow down the rate of disease progression ^11^; thus it appears most efficient to target the COPD-specific pathomechanisms at the earliest distinguishable state, when the extent of irreversible damage is still small, and their molecular processes are not yet convoluted with secondary processes and comorbidities, e.g., bacterial and viral infections, as they occur during the exacerbations typical for later stages of COPD. Since smoking cessation/replacement appears to be the most efficient therapy in smoking-related COPD ^11^, the models of early onset COPD can also be expected to be valuable tools for the development and testing of reduced risk products that may prevent COPD progression in a comparable manner as cessation does.

The networks reported here were created first from a literature scaffold and expanded via data enhancement using RCR (Phase 1), then they were made available online to the entire scientific community for critical review during the Network Verification Challenge (NVC) “Open Phase” (Phase 2) under the umbrella of the systems biology verification (sbv) IMPROVER project ^20^ ( Figure 1). Finally, a prioritized subset of 15 of these networks was discussed during an in-person jamboree meeting where the crowd-submitted revisions were reviewed and decisions to improve the networks were finalized (Phase 3). The final versions of the networks are available at https://bionet.sbvimprover.com for the public to view, and for registered users in the NVC to continue to discuss.

A variety of COPD networks have been created by various research groups, including networks focused on muscle to study skeletal muscle abnormalities ^21^, networks to compare COPD and asthma ^22^, and a knowledge management framework to integrate COPD clinical and experimental data ^23^. To our knowledge, this is the first set of crowd-verified networks available to the broader scientific community as a unified collection on a freely accessible web-based platform. Ultimately, this interface will allow for continuous input and improvement in the networks, leading to better understanding, diagnosis, and treatment of COPD.

## Methods

### Results

Original networks, NVC networks and COPD data sets used in: Enhancement of COPD biological networks using a web-based collaboration interface Original networks, NVC networks and their descriptions. The file contains the names of the original networks (as they were published), agglomerated NVC networks (as presented on the Bionet website), and network descriptions. The 15 networks that were discussed during jamboree are indicated by “X” in the column Discussed in Jamboree.
COPD data sets, their descriptions, and the comparisons used to build the COPD models during Phase 1. Reverse causal reasoning was performed using COPD and emphysema data sets from lung, small airway, and alveolar macrophages of early COPD patients and healthy smokers. Data Sets, the Gene Expression Omnibus (GEO) used to build the COPD networks. SCs, state changes defined using differentially expressed genes that meet the following criteria: FDR adjusted p<0.05, fold change ≥1.3, and minimum expression of 100 (for Affy platforms). HYPs, mechanisms or hypotheses predicted from the SCs and the Selventa Knowledgebase [1] with the following cutoffs: richness p<0.1, concordance p<0.1.
Early COPD was defined as Global Initiative for Chronic Obstructive Lung Disease (GOLD) stages 1 and 2. The three small airway data sets were merged using ComBat [2] because of the small sample size of early COPD patients within each data set. Lone emphysema is defined in the GSE10006 data set as patients who have normal spirometry but decreased transfer factor and evidence of emphysema on chest computed tomography scans. The lone emphysema data were selected because they might be useful in understanding COPD onset.
References 1. Catlett NL, Bargnesi AJ, Ungerer S, Seagaran T, Ladd W, Elliston KO, Pratt D: Reverse causal reasoning: applying qualitative causal knowledge to the interpretation of high-throughput data. BMC bioinformatics 2013, 14:340. 2. Chen C, Grennan K, Badner J, Zhang D, Gershon E, Jin L, Liu C: Removing batch effects in analysis of expression microarray data: an evaluation of six batch adjustment methods. PloS one 2011, 6:e17238.

***Phase 1: COPD enhancements using data and literature***

**Figure 3.**
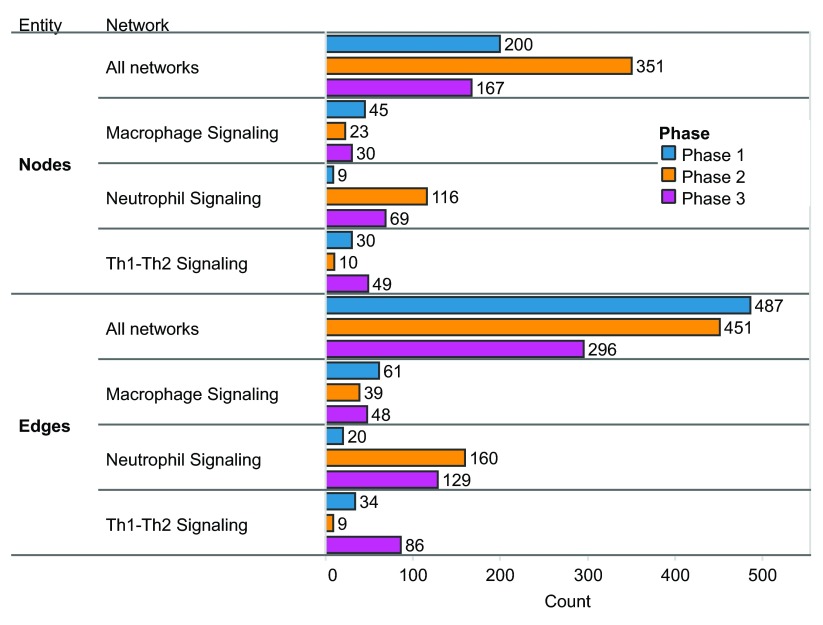
Nodes and edges added in each phase of COPD network construction. Summary of nodes and edges added to all networks and to three example networks in each phase. **A**) Nodes added in each phase. **B**) Edges added in each phase.

Ninety non-diseased lung networks published previously in the areas of cell proliferation, cell stress, inflammation, DNA damage, cell death, tissue repair, and angiogenesis were used as the initial scaffolds for COPD enhancement during Phase 1 ^3– 7^. Biological pathways implicated in COPD disease pathophysiology, including B-cell and T-cell activation, airway remodeling, extracellular matrix (ECM) degradation, efferocytosis, mucus hypersecretion, and emphysema were all captured within the modified network models. In total, 200 new nodes and 487 new edges were added: 415 of the edges were added to incorporate COPD mechanisms implicated in the literature, and 72 edges were added to incorporate 100 mechanisms predicted from COPD data by RCR to be relevant to COPD ( Figure 3). Because the models were built to represent COPD in humans, human evidence was preferred and made up the majority of the networks (74%).

During Phase 1, the networks with the most significant number of COPD enhancements in terms of percentage of the network with new nodes were the *Mucus Hypersecretion* (44%), *Th2 Signaling* (37%), *Macrophage Activation* (28%), *Fibrosis* (25%), *Autophagy* (11%), and *Apoptosis* (5%) networks. Networks that were not enhanced with COPD-specific mechanisms from the literature or RCR included the *DNA Damage* and *Notch Signaling* networks. Although both these networks relevant to the development of COPD, they were not augmented beyond the original, non-diseased network scaffolds, because no studies on the differences in signaling between non-diseased and diseased states were available. Although there may be papers that report on the correlation between COPD and these processes, network model building requires mechanistic information that will provide causal links within the model.

***Phase 2: Networks enhanced with lung- and COPD-relevant mechanisms by the crowd during the open phase***

Prior to deploying the COPD-enhanced biological networks on the NVC website for verification by the scientific community, the set of 90 networks was agglomerated by the model-building expert team to yield a more concise set of 50 networks that combined and standardized related/complementary cellular pathways (See Methods for details). For example, a new “Th1 Signaling” network model was created by merging three of the original networks that were relevant to the functional biology present in T-helper 1 cell populations: *Th1 Differentiation*, *Th1 Response*, and *T-cell Recruitment and Activation*. For a list of the original models that correspond to the agglomerated models and a description of the new models, see Dataset.

During Phase 2, a global community of scientists participated in the NVC by contributing their expertise to one or several of the network models. Scientists could contribute by verifying existing evidence for network edges using a system that allowed users to vote on evidence to indicate agreement or disagreement with its appropriateness within the network structure and boundary conditions. Participants were also encouraged to add new mechanistic biology in the form of network edges. In total, the 50 network models received 2456 evidence votes, 1795 of which supported the confirmation of evidence and 661 that favored the rejection of evidence (see Dataset). The *Neutrophil Signaling* network model received the largest share of voting activity, with 241 total votes or approximately 10% of all votes cast. Other network models that received large shares of the votes included the *Macrophage Signaling* (180 votes) and *Th1 and Th2 Signaling* network models (105 votes) (see Dataset). In addition to verifying existing literature evidence supporting edges in the network models, NVC participants could add novel biological information in the form of new literature evidence (for an existing edge) or contribute new network edges to incorporate new biological components into the network structure. In this way, the community of participants collectively contributed a significant amount of new information into the networks; among the 50 network models, a total of 885 new pieces of evidence, 351 new nodes, and 451 new edges were added ( Figure 3).

***Phase 3: Jamboree discussion and final decisions for next version networks***

Following Phase 2, a jamboree (Phase 3) was organized for a group of invited participants to discuss the network enhancements submitted by the crowd. To represent the crowd community, the top 20 active performers who created the most pieces of evidence and submitted at least 20 votes during the NVC were invited to an in-person jamboree to discuss network refinements as a group. Additional subject matter experts in the network biology, COPD, lung biology, and biological processes represented by the networks were invited to participate in the discussions and contribute their expert feedback independent from the network-building experts. Among the 50 network models evaluated during the online NVC, 15 were prioritized and selected for discussion during Phase 3 based on the level of crowd-sourced activity and their importance in COPD onset as considered by the network-building experts (see Dataset). The goal of Phase 3 was to provide an additional layer of “verification” for the online enhancements and to provide holistic comments on the network models at the molecular/biological entity level. In doing so, the three network models that had received the largest amounts of crowd activity ( *Neutrophil Signaling*, *Macrophage Signaling*, and *Th1 Signaling*) also underwent significant additional enhancements to improve granularity with respect to COPD onset and pathogenesis. In total, 167 nodes and 296 edges were added among all the network models reviewed during the jamboree sessions, and the three inflammatory networks received 89% of the nodes and 89% of the edges (148 nodes and 263 edges) ( Figure 3). Many of these changes came from the identification of missing mechanistic details of processes that occur in COPD (e.g. chemotaxis mechanisms in the *Macrophage Signaling* network model described in the examples in the “Macrophage signaling” section below).

**Figure 4.**
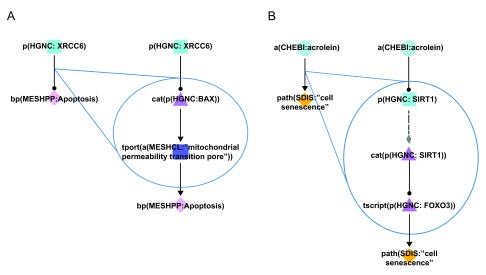
Improvements in the granularity of two representative network pathways. Cyan squares represent abundances, triangles activities, purple squares the movement of abundances from one cellular location to another, and diamonds biological processes. During Phase 3 of COPD network construction, improvements were made by adding mechanistic details to over-simplistic edges. **A**) In the *Apoptosis* network model, the original connection (left) simply indicated that XRCC6 decreased the process of apoptosis. The improved pathway connection (right) indicates that XRCC6 decreases the activity of BAX, which normally functions to facilitate the transport of calcium ions through the mitochondrial pores and thereby increases apoptosis. **B**) In the *Mechanisms of Cellular Senescence* network model, the original connection (left) simply indicated that acrolein increased the process of cellular senescence. The improved pathway connection (right) indicates acrolein mediates its effects on senescence via the activity of SIRT1 and the FOXO3 transcription factor. Triangle denotes activity, diamond denotes biological process or pathology, circle denotes abundance, rounded square represents transport, and square denotes protein abundance nodes. Solid edges denote causal relationships, dotted edges denote non-causal relationships such as a protein connected to its own activity.

In addition to adding mechanistic details of processes that occur in COPD, enhancements were incorporated to improve the granularity and connectivity within the network structures. In several instances, the improvements involved the creation of more detailed linear pathways connecting biological components. In one example, in the *Apoptosis* network model, the original network pathway indicated that the X-ray repair complementing defective repair in Chinese hamster cells 6 (XRCC6) protein decreased the process of apoptosis ^24^. During the Phase 3 discussions, additional literature evidence provided a more detailed mechanistic understanding of this phenomenon: XRCC6 was reported to decrease the activity of the BCL2-associated X protein (BAX) protein, which is known to increase mitochondrial permeability and therefore promote apoptosis ( Figure 4A). The overall effect of the negative regulation of BAX by XRCC6 was therefore a decrease in apoptotic cell death ^25^. By improving the granularity of this pathway in the *Apoptosis* network, a more comprehensive representation was achieved for components that are related to critical cellular processes mediating disease onset.

A similar improvement was incorporated into the *Mechanisms of Cellular Senescence* network model: the original network pathway indicated that the chemical acrolein (a common component of cigarette smoke) increased cell senescence ^26^. During Phase 3 discussions, the pathway connecting these two components was expanded using additional literature evidence. In several studies, acrolein was found to decrease the activity of sirtuin 1 (SIRT1), which is a known negative regulator of the forkhead box O3 (FOXO3) transcription factor, and FOXO3 activity is known to promote cellular senescence ( Figure 4B) ^26– 28^. Therefore, the overall observed effect was acrolein acting to potentiate cellular senescence in exposed cells, which is a well-characterized mechanism of action for this toxic chemical. Again, the generation of more comprehensive network models of biological processes in close proximity to disease onset allowed for a greater mechanistic understanding of how environmental factors can contribute to COPD development.

## Exemplary outcomes of the three-phase COPD network building process

### Th1 and Th2 signaling

As part of the pulmonary inflammatory process network building ^6^, five networks ( *T-cell activation and recruitment, Th1 differentiation, Th2 differentiation, Th1 Response, Th2 response*) were built to describe Th1 and Th2 signaling in the non-disease lung context. As described previously, during the preparation phase to NVC, two networks were built around the Th1 and Th2 cells.

***Phase 1: COPD augmentation of T-helper cell networks***

**Figure 5.**
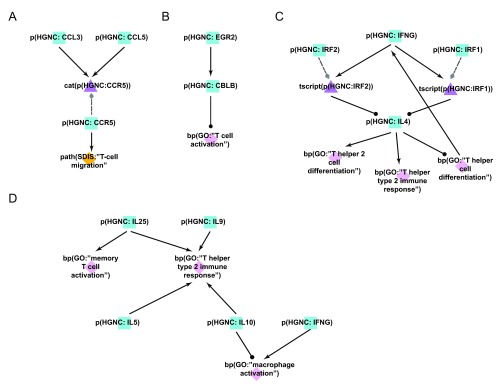
Enhancement of the T-cell networks during COPD network construction. **A**) During the literature-based COPD enhancement process in Phase 1, the protein CCL3, important for leukocyte migration and activation of T-cells, was added to the T-cell networks. **B**) During the open phase in Phase 2, the negative regulation of EGR2 on T-cell activation is a mechanistic detail that was added by the crowd. Overexpression studies demonstrated that EGR2 increased the activity of the E3 ubiquitin ligase CBL-B, which subsequently inhibited T-cell activation. **C**) During the jamboree discussions in Phase 3, the IFNG/IL-4 feedback loop mediating differentiation of Th1 vs. Th2 cellular subtypes via the activities of IRF1 and IRF2 was added to the new *Th1-2 Signaling* network model. **D**) During the jamboree discussions in Phase 3, the T-helper cell-produced chemokine effect on immune cells (e.g. IL-25 activates memory T-cells) was added to the new *Th1-2 Signaling* network. Triangle denotes activity, diamond denotes biological process or pathology, and square denotes protein abundance nodes. Solid edges denote causal relationships, dotted edges denote non-causal relationships such as a protein connected to its own activity.

Mechanisms that describe T-cell activation and recruitment induced by neutrophils, macrophages, and dendritic cells were added to the T-cell networks during Phase 1. These immune cells secrete various chemokines that were reported to recruit T-cell populations (i.e. CD8+ cytotoxic T-cells) to injured tissue in an acute inflammatory state ^29^. Alveolar macrophages secrete interleukin 15 (IL15), which is capable of activating both the interleukin 2 (IL2) and IL15 receptors on T-cells and acts as a potent inducer of cell migration to the lung. Dendritic cells within the lung play an important role in this process by secreting chemokine (C-C motif) ligand 3 (CCL3) in response to cigarette smoke, which helps recruit CD8+ T-cells to the lung ^29^. Chemokine (C-C motif) receptor 5 (CCR5) is the receptor for CCL3 and its presence in the lung has been shown to correlate with the severity of COPD ^30^. CCL3 is one example of a node that was added during the literature-based COPD enhancement process in Phase 1 ( Figure 5A). Many of the disease-relevant mechanisms identified in the literature curation phase were corroborated by mechanisms predicted from COPD-relevant data sets using RCR (see Methods), including T-cell activation mechanisms (CD28 molecule (CD28) and T cell receptor beta locus (T\RB), and chemokines and cytokines that activate and are secreted by T-cells (chemokine (C-C motif) receptor 3 (CCR3), CCR5, IL2, interleukin 4 (IL4), interleukin 6 (IL6), interleukin 10 (IL10) and interleukin 13 (IL13)). The prediction of these mechanisms in COPD data sets showed that T-cell activation and migration in response to smoke-exposed lung represents an important process in the innate immune response. In total, 30 nodes and 34 edges were added to the Th1 and Th2 networks during the internal COPD enhancement process.

***Phases 2 and 3: T-cell network crowd improvements***

During the open phase (Phase 2), the Th1 and Th2 networks received 105 votes from the scientific community, as well as 10 new nodes, 9 new edges, and 13 new pieces of evidence. One such addition to the *Th1 Signaling* network was the regulatory influence of early growth response 2 (EGR2) on T-cell activation; the submitted evidence demonstrated that overexpression of EGR2 promoted increased activity of the E3 ubiquitin ligase CBL-B and subsequent inhibition of T-cell activation ^31^ ( Figure 5B).

During the Phase 3 jamboree sessions, the group decided to combine the individual *Th1 Signaling* and *Th2 Signaling* networks into a single, unified network model titled *Th1-Th2 Signaling* to better represent the interplay between the T-helper cell populations *in vivo.* It was also decided to add granularity to transcriptional pathways mediating Th1 versus Th2 cellular activation and differentiation; one example was the addition of two transcription factors, interferon regulatory factors 1 and 2 (IRF1 and IRF2), that are known to act downstream of interferon-gamma (IFNG) to suppress IL4 expression in Th2 cell populations ^32^. IFNG is secreted by Th1 cells and this pathway potentiates Th1 responses while suppressing Th2 responses in the tissue. The addition of this feedback mechanism during Phase 3 contributed to a more comprehensive network describing the interactions between Th1 and Th2 cells ( Figure 5C). Further network enhancements discussed in the jamboree largely emphasized the downstream effects of T-helper cells in potentiating inflammatory signaling by activating additional immune cells in a disease context. For example, secretion of IL5 activates eosinophils, whereas secretion of IL10 and IFNG activates macrophages in the diseased tissue ^33– 35^. This interplay between immune cell populations was incorporated into the new *Th1-Th2 Signaling* network model and better captures the signaling interconnectivity present during disease development ( Figure 5D). In total, 12 new nodes and 28 new edges were added to the *Th1-Th2 Signaling* network model during the jamboree discussions, thereby creating a more comprehensive biological network of T-helper cell activity and their interactions with other immune cells in the context of COPD.

### Macrophage signaling

As part of the pulmonary inflammatory process network building ^6^, three networks ( *Macrophage Differentiation, Macrophage Activation, and Macrophage-mediated Recruitment of Neutrophils*) were built to describe macrophage biology in the non-disease lung context. During the preparation phase to NVC, these three networks were merged to obtain an overall picture of macrophage biology.

***Phase 1: COPD augmentation of macrophage networks***

**Figure 6.**
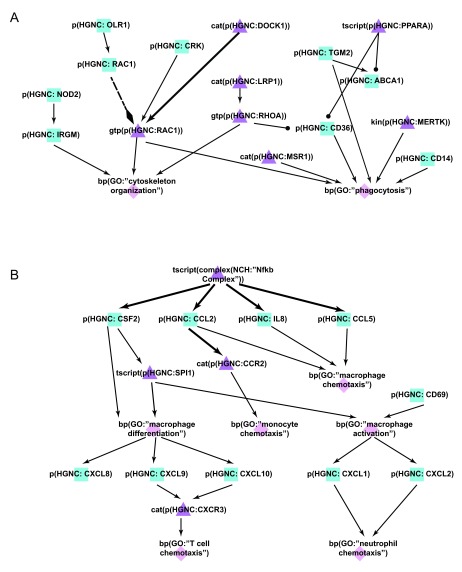
Enhancement of the macrophage networks during COPD network construction. **A**) During the literature-based COPD enhancement process in Phase 1, efferocytosis mechanisms were added to the macrophage networks to take into account its dysregulation effect in COPD. **B**) During the jamboree discussions in Phase 3, chemotaxis and differentiation mechanisms were identified and subsequently added to the latest version of the *Macrophage Signaling* network. Triangle denotes activity, diamond denotes biological process, and square denotes protein abundance nodes. Solid edges denote causal relationships, dotted edges denote non-causal relationships such as a protein connected to its own activity.

Macrophages play roles in many COPD disease processes such as clearance of apoptotic neutrophils, tissue destruction, and recruitment of other immune cells by their secretion of cytokines ^36^. Macrophage signaling mechanisms were added to the network in Phase 1, with a focus on components related to efferocytosis ( Figure 6A). Efferocytosis is a well-conserved mechanism for the phagocytic removal of apoptotic cells by innate immune cells, such as macrophages, and the process is critical for the resolution of inflammation via the removal of dying cells and antigenic cellular debris. Phagocytically impaired macrophages have been shown to display decreased expression of peroxisome proliferator-activated receptor gamma (PPARy) and efferocytosis-specific bridge molecules, such as growth arrest-specific 6 (GAS6) and milk fat globule-EGF factor 8 protein (MFGE8) ^37^. The number of apoptotic cells was shown to increase in COPD because of exposure of lung tissue to toxic chemicals present in cigarette smoke; for example, and their accumulation was exacerbated by the simultaneous smoke-induced impairment of the phagocytic ability of alveolar macrophages ^38^. Apoptotic cells exhibit surface changes that distinguish them from viable cells, and these changes were recognized by efferocytic receptors including CD36 molecule (CD36), CD14 molecule (CD14), and Stabilin-1/2 (STAB1:STAB2) ^39^. Reduced efferocytosis observed in COPD because of oxidant-driven and Rho-mediated inactivation increased the likelihood of aberrant antigen exposure from apoptotic cells, thereby perpetuating the chronic inflammatory state that is a hallmark of COPD ^40– 42^. In adding efferocytosis mechanisms to the macrophage network, we focused on the surface receptors and bridge proteins such as CD36 and GAS6. In total, 45 nodes and 61 new edges were added to the macrophage model during the internal COPD enhancement phase.

***Phases 2 and 3: Macrophage network crowd improvements***

During the open phase (Phase 2), 180 total votes were cast for network evidence, with 23 new nodes and 39 new edges added by the crowd. In addition, 72 new pieces of evidence were contributed to support pre-existing edges in the network. The surfactant protein A1 (SFTPA1), which was observed to be increased in COPD ^43^, was added to the network. Its effect on macrophages of increasing interleukin-1 receptor-associated kinase 3 (IRAK3) and interleukin 1, beta (IL1B) were also added to the network during the open phase. Granularity enhancements around IFNG and nucleotide-binding oligomerization domain containing 2 (NOD2), both components of inflammatory signaling, were also added to augment the network models with causal relationships proximal to COPD.

During the Phase 3 jamboree discussions, several network enhancements were made in macrophage chemotaxis and differentiation ( Figure 6B). Within the chemotaxis process, the nodes chemokine (C-C motif) ligand 2 (CCL2) binding to chemokine (C-C motif) receptor 2 (CCR2) and leading to macrophage chemotaxis were added. The CD69 molecule (CD69) associated with macrophage activation by cigarette smoke was also added. In addition, the effects of activated macrophages on other immune cells were expanded within the network model, including chemokine (C-X-C motif) ligand 1 (CXCL1) and chemokine (C-X-C motif) ligand 2 (CXCL2) leading to neutrophil chemotaxis, and chemokine (C-X-C motif) ligand 9 (CXCL9) and chemokine (C-X-C motif) ligand 10 (CXCL10) binding to CXCR3 and leading to T cell recruitment. In total, 30 new nodes and 48 new edges were added to the *Macrophage Signaling* network during Phase 3, thereby providing a more comprehensive network of macrophage activation and its effect on other immune cells active in COPD.

### Neutrophil signaling

As part of the pulmonary inflammatory process network building ^6^, two networks ( *Neutrophil Response* and *Neutrophil Chemotaxis*) were built to describe neutrophil biology in the non-disease lung context. During the preparation phase to NVC, these two networks were merged to constitute the *Neutrophil Signaling* network.

***Phase 1: COPD augmentation of neutrophil networks***

**Figure 7.**
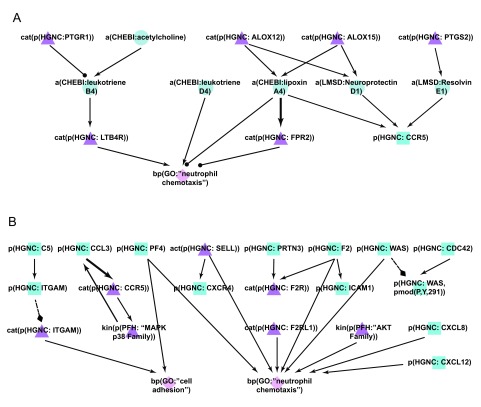
Enhancement of the neutrophil network during COPD network construction. **A**) During the literature-based COPD enhancement process in Phase 1, lipids and their effects on neutrophil chemotaxis were added to the new *Neutrophil Signaling* network. **B**) During Phases 2 and 3, neutrophil adhesion and chemotaxis mechanisms were added to the new *Neutrophil Signaling* network. Triangle denotes activity, diamond denotes biological process, circle denotes abundance, and square denotes protein abundance nodes. Solid edges denote causal relationships, dotted edges denote non-causal relationships such as a protein connected to its own activity.

During Phase 1, the *Neutrophil Signaling* network was enhanced primarily with components related to lipid-response pathways. In response to lung damage, leukocytes and tissue-resident cells were reported to interact to generate lipid mediators that enhance the airway immune response and engage defense mechanisms ^44^. Neutrophils, endothelial cells, and macrophages generate prostaglandins and leukotrienes from arachidonic acid during the initial inflammatory response, which amplifies the inflammation signals in the local area and potentiates the process of tissue destruction ^45^. Subsequently, the prostaglandins PGE2 and PGD2 are generated in a cyclooxygenase-dependent way to promote synthesis of lipid mediators with anti-inflammatory activity, such as the lipoxins. Lipoxins inhibit neutrophil recruitment to inflamed sites and suppress their pro-inflammatory actions, but promote recruitment of macrophage precursors ^46^. Lipoxin A4 stimulates macrophages to phagocytose apoptotic neutrophils, and resolvins and protectins, which represent another class of lipid mediators, activate anti-inflammatory pathways and stimulate clearance of inflammatory infiltrates by macrophage phagocytosis ^47– 49^. In total, 9 nodes and 20 edges were added to the network model including lipid mediators such as lipoxin A4, resolvin E1, and neuroprotectin D1 ( Figure 7A).

***Phases 2 and 3: Neutrophil network crowd improvements***

The *Neutrophil Signaling* network was the network most edited by the crowd during the open phase, with the addition of 116 new nodes, 160 new edges, 181 new pieces of evidence, and 241 votes cast. The new edges described neutrophil chemotaxis including new nodes like platelet factor 4 (PF4) and protease-activated receptor 2 (F2RL1). Chemokines such as chemokine (C-X-C motif) ligand 8 (CXCL8) and chemokine (C-X-C motif) ligand 12 (CXCL12), and members of the serine/threonine kinase (AKT) family that have also been shown to induce neutrophil chemotaxis were added to the network ( Figure 7B) ^50^.

Following the jamboree discussions, additional signaling that described cytoskeletal and adhesion mechanisms necessary for neutrophil chemotaxis, and additional neutrophil activation mechanisms, were incorporated in the new *Neutrophil Signaling* network ( Figure 7B). The role of the CDC42-WASp complex in regulating neutrophil chemotaxis at the cytoskeletal level was incorporated ^51^, as well as other mechanisms of neutrophil chemotaxis including the role of the complement component 5 (C5) in regulating integrin, alpha M (ITGAM) ^52^, and the role of CCL3/CCR5 in stimulating neutrophil migration ^53^. In all, 69 nodes and 129 edges were added. The new mechanisms that were incorporated into the *Neutrophil Signaling* network added significant granularity to the neutrophil chemotaxis process, which is a key driver of the inflammatory cascade that promotes the development of COPD.

## Discussion

Here we report the construction of a COPD-enhanced network model set using a novel methodology that combined traditional manual literature curation and data-driven approaches with a global crowdsourcing endeavor to generate the most comprehensive representation of biological phenomenon proximal to the onset of COPD that is available to date. The three phases of network construction each contributed in different ways to building a more comprehensive network. The Phase 1 literature and data-driven enhancement of the already existing non-diseased networks resulted in the addition of COPD biomarkers and disease drivers known to be associated with COPD, while the Phase 2 crowdsourcing largely focused on contributions to cell-specific networks, and the Phase 3 jamboree discussions uncovered missing signaling processes relevant to COPD.

### COPD biomarkers and processes added to non-diseased networks

During Phase 1, the non-diseased networks were expanded within the COPD context by the addition of biomarkers, disease drivers, and processes that were reported to increase in COPD, as well as mechanisms predicted in COPD data sets. Most of the edges added to the networks were lung relevant but not specifically investigated in a COPD background. Because of the limited number of mechanistic studies in COPD models that have been published, network construction was focused on adding COPD-known processes and biomarkers in tissue and experimental contexts relevant for COPD (lung, smoking) to the existing non-disease networks.

Modeling the process of efferocytosis is an example of the addition of COPD processes to the non-disease networks. The efferocytosis process of phagocytic uptake of apoptotic cells by macrophages is frequently disrupted in COPD tissue, and this disruption is thought to potentiate the chronic state of inflammation in the diseased lung ^40– 42^. A new network model detailing components related to efferocytosis was constructed from information available in the published literature with the majority of edges coming from general macrophage experiments. Th2 activation cascades and macrophage signaling events were also implicated generally in the context of COPD, and therefore the non-diseased network models were enhanced by the addition of these pathways from lung-relevant studies. Network models detailing other processes not widely implicated in COPD, such as DNA damage and Notch signaling, which are more generalized conserved biological phenomenon, received very few, if any, enhancements during the COPD literature curation phase.

In addition to adding COPD processes during Phase 1, we also added COPD biomarkers and mechanisms predicted by RCR to be active in COPD data sets. Biomarkers associated with COPD included chemokines, cytokines, matrix metalloproteinases (MMPs), and other matrix degradation products. Examples of cellular mechanisms uncovered by the data-driven approach included the cytokines IL19 and IL3, as well as the serine protease inhibitor SERPINA1. IL3 is a growth-stimulating cytokine for many inflammatory cells, including macrophages, and IL19 is produced by monocytes and activates the inflammatory STAT3 pathway in several cell types. SERPINA1 is a potent elastase inhibitor, the presence of which plays a critical role in controlling the protease cascade leading to tissue destruction and emphysema. Overall, the RCR approach yielded a diverse range of biological features that were incorporated among a large percentage of the network models, thereby broadening the scope of many networks to include components with potential connections to disease that have not been investigated previously in the COPD context.

### Crowdsourcing efforts focused on cell-specific networks

During the NVC, scientists from around the world browsed the publically available networks on a website, voted on and submitted new evidence, and created new nodes and edges. As may have been expected, several of the more well-studied processes in the literature (e.g. NF-kB pathways leading to inflammatory signaling) attracted a great deal of voting activity within the networks and primarily corroborated known biology. However, participants were incentivized to create new evidence to support existing edges based on the large number of points received by them for this activity. It was this aspect of the challenge that truly demonstrated the power of crowdsourcing because, in many instances, the community of users located lung-relevant and/or more recent publications to better support the existing network architecture and improve the overall relevance of the network models to COPD. With nearly 900 new pieces of evidence (from 479 unique PMIDs) added by the challenge crowd, a significant overall enhancement of the networks was achieved in a relatively short time (5 months), which demonstrated the remarkable utility of harnessing knowledge from the global scientific community for a specific application. Specifically, 30% (266/885) of all the new pieces of evidence and 46% (208/451) of all the new edges that were contributed fell within three network models, namely the *Neutrophil Signaling, Macrophage Signaling*, and *Th1-Th2 Signaling* networks. These networks were edited more than other networks because of their clear boundaries, which allowed scientists to narrow their search to a particular cell type. Networks such as *Clock, Wnt*, *mTor*, and *Regulation of CDKN2A expression* were edited minimally and received more ‘Down’ votes than the cell-specific networks, possibly because of the more ambiguous boundaries of which cell types could be included. This observation emphasizes the need for clear boundaries in a crowdsourcing effort. In the case of general networks such as *Cell Cycle, Response to DNA Damage*, ** and *Oxidative Stress*, many experiments concerning these processes have been performed in cell types that were excluded in our boundaries (i.e. tumorigenic cell lines). Perhaps boundary conditions could be loosened for networks such as these if it is assumed that signaling is conserved across different cell types.

### Jamboree discussions identified missing processes relevant to COPD

The final phase of network improvements emphasized the discussion and consolidation of all submissions from the challenge crowd to synthesize more holistic changes within the set of network models. During the challenge, participants worked individually on the website adding individual edges, but did not have the ability to make major changes to the structure of the network models. The in-person jamboree discussions were therefore an opportunity to implement broader changes to better represent the biological processes as they related to COPD. These discussions were led by experts in the subject matter of the processes that the networks represented. During these sessions, missing pieces of biology and the interactions of different cell types in COPD were identified. In this manner, the jamboree was very conducive to broader network structural changes that made the set of network models more informative and representative of processes implicated in COPD and, therefore, more useful to a broader group of scientists.

### Unique features of the collaborative networks

In recent years, crowdsourcing has emerged as a powerful tool to address topics related to “big data” in the domain of the life sciences, particularly in topics related to systems biology. For example, the series of DREAM challenges empowered the global scientific community to build application-specific, clinically relevant predictive biological networks using vast quantities of genomic data ^54^. Similarly, the recent sbv IMPROVER challenges allowed researchers to participate in collaborative competitions to validate systems biology research, for example, by testing and validating computational approaches that are used to classify clinical samples based on transcriptional data ^55– 57^. In the current approach, we describe a unique paradigm for biological network construction that combines a predictive computational methodology with a large-scale crowd sourcing approach to generate very comprehensive network models describing COPD pathogenesis.

Compared with other published COPD networks, the networks described here are more comprehensive in scope, are focused on molecular pathways that can drive disease rather than on descriptions of more general clinical or physiological measures, and have been improved using crowdsourcing ^21– 23^. The Synergy-COPD European project is similar in its goal of creating a model of COPD for better understanding of the disease by combining information from many different sources. However, Synergy-COPD comprises seven physiological-focused mathematical networks rather than the 50 molecular networks described here, and does not currently have an intuitive web interface that allows users to freely navigate the resulting networks ^23^.

Compared with other more general pathway approaches such as KEGG ^1^, the networks we describe contain edges that have one or more detailed evidences supported by a specific literature reference and contain tissue and species-level metadata. In our approach each of these pieces of evidence under an edge can be validated with the potential for a larger crowd with wide expertise, compared to a non-crowdsourced approach where the small group constructing the networks may not be able to sufficiently cover all the expertise necessary to verify every pathway within these networks. The BEL language syntax allows many participants to contribute by standardizing the biological representation and requiring that each node be associated with a namespace, which standardizes the representation of gene names and biological processes. The comparison of our network models with other resources has been described in other articles ^58, 59^ and in a book chapter ^60^.

The web-based platform captures network provenance, allowing for a transparent record of what has been validated with a full revision history ^58^. The uncertainty for specific edges based on voting patterns can be demonstrated with the full voting history being captured in the network versions. By incorporating a continuous “feed” of real time enhancements submitted on the website, users are able to view the most up-to-date networks at any time; network models created using other platforms not available for crowdsourced editing remain static representations of biology and frequently do not include the most recent findings from the scientific literature. Currently networks with the most recent crowd edits can be viewed, but not downloaded. Networks with changes from the most recent Jamboree meeting are made available for download.

Another novel component of these networks is the incorporation of RCR predictions to enhance the overall biological representation within the network models. RCR analysis was performed on human COPD gene expression data sets in the public domain in order to predict potential mechanisms implicated in COPD onset and include as nodes in the networks. This unbiased approach resulted in the addition of many new nodes among the networks predicted to be active based on COPD gene expression footprints that may have less well-established or direct connections to disease etiology. As such, this important aspect of network construction potentially captures those biological components that may have “emerging” roles in disease progression. The iterative nature of the network enhancement process facilitated by the Bionet platform allows for new biology and supporting evidence to be incorporated into the networks as new findings emerge in the literature and therefore generate the most comprehensive, up-to-date COPD model sets available to the scientific community.

The utility of the resulting networks and to further analyze the crowdsourcing process itself can be assessed by evaluating the impact of the changes on the analyses we have published previously ^60– 67^. Moreover, an extensive analysis leveraging multiple relevant datasets will be conducted and the results will be published.

The enhanced crowd-verified models are publicly available on the sbv IMPROVER website ( https://bionet.sbvimprover.com/) and remain open to receive further enhancements from the online community. Because the first iteration of the NVC proved the effectiveness of this approach and because the networks can continue to be reviewed by the crowd, a second iteration of the NVC (NVC2) has been started so that additional modifications and recently published literature can be incorporated. This will help to continually refine the network models and strengthen the relevance to the processes that underlie the development of COPD. The crowd verification approach continues to be refined, so, in addition to disease process-centered networks, other networks including chemical-centered networks can be built using a similar approach. These networks can aid in the development of more efficient interventions and enhance toxicological assessment of environmental exposures that may also contribute to the development of COPD.

## Conclusion

Here we describe a novel approach to biological network construction and have generated a suite of COPD-relevant network models that the larger scientific community is free to edit and explore. Networks are available for download from the sbv IMPROVER website ( https://bionet.sbvimprover.com/) upon registration and taking a certain number of actions as a participant (e.g., voting on an evidence). Scientists from all backgrounds are encouraged to submit additional network enhancements as participants in the NVC2 ^68^. By building the network model set in the BEL language format, we have generated a model framework suitable for biomarker discovery and for the interpretation of transcriptomic signatures ^59– 66^. More generally, this large assembly of biological knowledge relevant to human lung will be of great use to both academic and industry users in promoting future research in this area of great therapeutic importance.

## Methods

### Phase 1: COPD enhancement using data sets and literature

Networks that described molecular mechanisms of five broad biological processes were constructed previously using a literature and data mining approach. These networks cover mechanisms of cell proliferation ^5^, cell stress ^4^, DNA damage, autophagy, cell death and senescence ^3^, pulmonary inflammation ^6^, and tissue repair and angiogenesis ^7^ in the non-diseased pulmonary context. To create COPD-relevant networks, these non-diseased networks were enhanced by incorporating COPD mechanisms sourced using a literature and data set approach ( Figure 1) in an iterative approach, as described in detail for the non-diseased network model construction, by a team of subject matter experts in computational biology, molecular biology, inhalation toxicology, and COPD.

### Boundary conditions

Because the goal of the research was to understand COPD onset, the focus of these networks was on early stage COPD mechanisms (Global Initiative for Chronic Obstructive Lung Disease (GOLD) stages I and II). When supporting literature from early COPD studies was not available, stage-independent COPD studies were used. When COPD studies were not found, the inclusion criteria were expanded to studies from non-diseased context, and mechanisms active in processes implicated in COPD were incorporated into the disease models. Literature describing the processes active in acute exacerbation in COPD patients was excluded from the supporting edges of the network models. In order to focus on the molecular mechanisms most specific to early stage COPD, we also excluded context from diseases with different pathogenesis and differential diagnosis: lung cancer and non-cancerous lung diseases, such as cystic fibrosis, acute respiratory distress syndrome, idiopathic pulmonary fibrosis, septic pneumonitis, obliterative bronchiolitis, pneumoconiosis, bronchiectasis, viral and bacterial infections, and, allergic responses/asthma, bronchitis. Animal inhalation studies with solid particles (e.g. titanium dioxide, quartz, asbestos, carbon black, and diesel exhaust) were also excluded due to their specific mode of action. Ideally, all nodes and edges of the network model would be supported by published data from experiments conducted in the tissues and cell types found in the lung under the conditions of early COPD, e.g., airway and alveolar epithelial cells, lung fibroblasts, resident and recruited immune cells, and microvascular cells. These were prioritized but the respective cell types were also considered from other tissue origin if such lung specific context was not reported in the literature. For *in vitro*-specific exclusion criteria, tumor-derived cell lines, immortalized cell lines, neuronal cells, and cell types that are not found in the respiratory/vascular system were excluded. In some cases, we made exceptions and included non-lung cell types for canonical mechanisms for which there was additional evidence from the literature that the relationship was not tissue-specific but could also take place in the lung. Human-specific connections were prioritized, but where human data were not available, knowledge has been augmented with orthologous causal assertions derived from rat and mouse sources included after homologization in the Selventa knowledgebase where human data were not available ^5^.

The 90 previously published non-diseased network models used for the initial substrate included networks involved in cell proliferation ^5^, cell stress ^4^, DNA damage, apoptosis, senescence, autophagy, necroptosis (DACS) ^3^, pulmonary inflammation (IPN) ^6^, and tissue repair and angiogenesis (TRAG) ^7^. The *Endothelial Shear Stress* network from the cell stress model was excluded because the focus of the COPD Network was to describe lung biology.

### Literature enhancement

We conducted a broad survey of the literature to locate studies that had investigated the mechanistic biology of COPD pathogenesis and processes involved in COPD. Potential COPD biomarkers from sputum, bronchoalveolar lavage, and mouse and human blood samples, and mechanisms that regulate COPD processes were gathered from the literature and curated. Because only a small number of the studies had focused on early COPD, we expanded our searches to include stage-independent COPD studies, but excluded late-stage processes. Some processes known to be closely linked to COPD pathogenesis (e.g. B-cell activation and T-cell recruitment to lung tissue) have not been studied directly in the disease context; however, literature that detailed cell-type-specific canonical biology was sourced irrespective of the disease context.

### Data enhancement

RCR was performed using Gene Expression Omnibus (GEO) COPD and emphysema data sets from lung, small airway, and alveolar macrophages of early COPD patients and healthy smokers (see Dataset) ^69– 73^. RCR has been used previously to predict upstream regulators from transcriptomic data ^8^. Mechanisms that were predicted by RCR to be active and that were not already incorporated in the non-diseased networks were vetted on an individual basis to locate supporting literature for their potential involvement in COPD pathogenesis. Mechanisms that had not been studied directly in a COPD context were evaluated in an expanded tissue context to consider tissue deemed disease-relevant (e.g. alveolar macrophages). Mechanisms that were deemed relevant were connected in the most appropriate network based on their probable roles in COPD or lung biology.

### Network agglomeration

To generate a more concise model set for presentation to the crowd during the NVC, we consolidated networks associated with related biological processes among the 90 COPD-enhanced networks. An example of this consolidation is the merging of three non-disease networks related to T-helper 1 cells ( *Th1 Differentiation, Th1 Response,* and *T-cell Recruitment/Activation*) into a single new *Th1 Signaling* network. Fifty-six of the original 90 networks were combined into a concise set of 16 network models; the remaining 34 networks remained as standalone network models (see Dataset), yielding a final set of 50 models that were posted on the NVC website for review by the scientific crowd. In addition to the network agglomeration, protein, gene expression, and secretion edges were agglomerated to reduce the number of edges required for verification.

### Phase 2: NVC Open Phase

The crowd verification process of improving biological networks has been published previously ^20^. Briefly, the full set of 50 COPD-relevant network models was posted on the BioNet web portal ^68^ for a period of 20 weeks (the “Open Phase”), during which time a global community of participants were invited to submit biological improvements to the models. The improvements included submission of new evidence, additional literature publications to support existing network edges, and submission of new biological edges with supporting evidence for relationships that were not represented in a network. Users could also vote on evidence to indicate agreement or disagreement with its appropriateness within the network structure; disagreements often indicated improper tissue or experimental context for the given network. Evidence that received at least four ‘Up’ votes was “locked” to indicate crowd approval and evidence that received at least four ‘Down’ votes was “locked” to indicate rejection by the crowd. Depending on the frequency and type of submitted improvements, participants received credit points and were assigned a dynamic ranking on the community Leaderboard. For more information about the NVC challenge, see the 5-minute overview videos at https://sbvimprover.com/challenge-3/videos or the 1-hour webinars at https://sbvimprover.com/challenge-3/tutorials.

### Phase 3: Jamboree meeting

When the open phase was closed, the top-ranked participants were invited to a 3-day-long in-person jamboree to discuss improvements submitted by the community and to further refine the network models. Subject matter experts in lung, COPD, and network biology, as well as experts in other related biological processes, were also invited to guide the discussions and to provide expert feedback of missing or misrepresented signaling. Scientists involved in the construction of the original non-disease networks and Phase 1-enhanced networks were present to provide feedback for the rationale behind the boundary conditions and the mechanics of network construction and BEL. During the jamboree, 15 networks were prioritized to discuss in small groups of 6–10 people focusing on one network at a time. At the end of each session, final decisions were made about follow-up actions for each network and these actions were carried out subsequently by the scientists who constructed the original networks because of their familiarity with the mechanics of network construction and BEL.

The changes to the 15 networks that were discussed during the jamboree are posted online ^68^ in open-source XGMML (eXtensible Graph Markup and Modeling Language) format.

### BEL: the language of the networks

**Figure 8.**
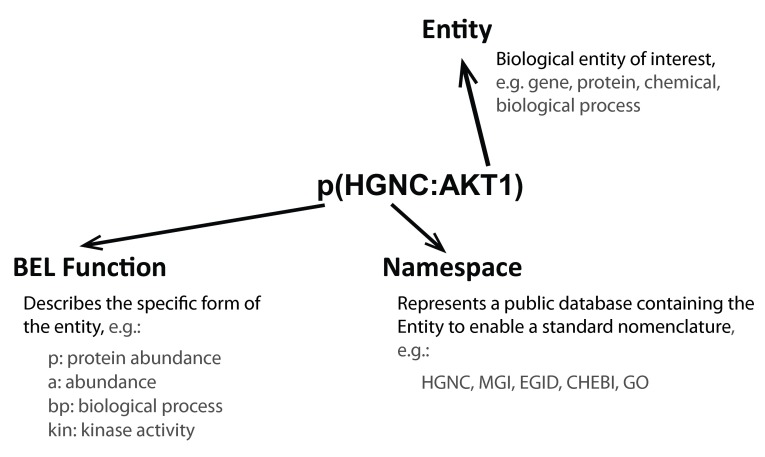
Structure of a BEL node. A BEL term is the standard way a node is described. It includes an entity that is described using standard nomenclature in the Namespace and the Function fields of the entity.

The networks were built using the Biological Expression Language (BEL), which is an open source language that can represent scientific findings in the life sciences in a computable form ^74^. BEL was designed to represent scientific findings by capturing causal and correlative relationships in context, where context can include information about the biological and experimental system in which the relationships were observed and the supporting publication citations. The structure of a BEL node, which includes the biological entity, the namespace or database to standardize the nomenclature of the entity, and the function that describes the type of entity (protein, chemical, biological process, family, complex, etc), is shown in Figure 8. Table 1 and Table 2 show the definition of the prefixes for BEL namespaces and functions that appear in the networks.

**Table 1.** BEL functions.

Prefix	Function
a	abundance
bp	biological process
cat	catalytic activity
sec	cell secretion
surf	cell surface expression
chap	chaperone activity
complex	complex abundance
composite	composite abundance
deg	degradation
fus	fusion
g	gene abundance
gtp	GTP bound activity
kin	kinase activity
m	microRNA abundance
act	molecular activity
path	pathology
pep	peptidase activity
phos	phosphatase activity
p	protein abundance
pmod	protein modification
rxn	reaction
ribo	ribosylation activity
r	RNA abundance
sub	substitution
tscript	transcriptional activity
tloc	translocation
tport	transport activity
trunc	truncation

**Table 2.** BEL namespaces.

Prefix	Namespace
EGID	Entrez Gene Identifiers
HGNC	HGNC Approved Gene Symbols
MGI	MGI Approved Gene Symbols
RGD	RGD Approved Gene Symbols
SPAC	Swiss-Prot Proteins (Accession Numbers)
SP	Swiss-Prot (Entry Names)
HGU95AV2	Affymetrix GeneChip Human Genome U95Av2
HGU133AB	Affymetrix GeneChip Human Genome U133AB
HGU133P2	Affymetrix GeneChip Human Genome U133Plus2
MGU74ABC	Affymetrix GeneChip Mouse Genome U74ABC
MG430AB	Affymetrix GeneChip Mouse Expression Set 430
MG4302	Affymetrix GeneChip Mouse Genome 430 2.0
MG430A2	Affymetrix GeneChip Mouse Genome 430A 2.0
RG230AB	Affymetrix GeneChip Rat Expression Set 230AB
RG2302	Affymetrix GeneChip Rat Genome 230 2.0
CHEBIID	Chemicals of Biological Interest (Identifiers)
CHEBI	Chemicals of Biological Interest (Names)
LMSD*	LIPID MAPS Structure Database (Names)
GOAC	GO Biological Processes (Accession Numbers)
GO	GO Biological Processes (Names)
MESHPP	MeSH Phenomena and Processes (Names)
MESHD	MeSH Diseases (Names)
MESHCL	MeSH Cell Locations (Names)
GOCCACC	GO Cellular Component (Accession Numbers)
GOCCTERM	GO Cellular Component (Terms)
PFH	Named Human Protein Families
NCH	Named Human Complexes
PFM	Named Mouse Protein Families
NCM	Named Mouse Complexes
PFR	Named Rat Protein Families
NCR	Named Rat Complexes
SCHEM	Selventa Legacy Chemical Names
SDIS	Selventa Legacy Disease Names

*Unofficial BEL namespace to be formalized in BEL 2.0

## Data availability

The data referenced by this article are under copyright with the following copyright statement: Copyright: © 2015 The sbv IMPROVER project team (in alphabetical order) et al.

Data associated with the article are available under the terms of the Creative Commons Zero "No rights reserved" data waiver (CC0 1.0 Public domain dedication). 

Up-to-date networks including all users’ activity can be browsed freely on the Bionet website ( https://bionet.sbvimprover.com/). Permanent URLs to each network are listed in the associated Data Set (Original networks, NVC networks and their descriptions). Networks can be downloaded by logged in users who had a few actions on the site as XGMML file for offline use in the version that started a verification phase, i.e. after review and QC by experts. The 15 networks discussed in the jamboree are available in a post-jamboree version. Moreover, different versions of the networks are available to browse and download in diverse formats from the CBN database available at causalbionet.com.

## Data availability

The data referenced by this article are under copyright with the following copyright statement: Copyright: © 2015 The sbv IMPROVER project team (in alphabetical order) et al.

Data associated with the article are available under the terms of the Creative Commons Zero "No rights reserved" data waiver (CC0 1.0 Public domain dedication). 

*Figshare:* Original networks, NVC networks and COPD data sets used in: Enhancement of COPD biological networks using a web-based collaboration interface http://dx.doi.org/10.6084/m9.figshare.1284583 ^75^
