# Supplementary material for: Enhancement of COPD biological networks using a web-based collaboration interface
Source: F1000Res. 2015 May 20;4:32. Originally published 2015 Jan 29. [Version 2] doi: 10.12688/f1000research.5984.2 (PMC4350443; doi:10.12688/f1000research.5984.2)
Supplement: Peer review history

# Peer review: Referee response for version 1

**DOI:** 10.5256/f1000research.6402.r7527

**Published:** 2015-03-02

**Authors:** Patrick Ruch

**Affiliations:** 1 BiTeM group, Library and Information Sciences, University of Applied Sciences—HEG, Geneva, Switzerland

I would welcome more information about the original networks, which were generated out of text/data mining. In particular, I would like to know what keywords were used to fetch the source articles (a set of genes/gene products, e.g. AQP5, MUC5...; a list of pathologies and synonyms, e.g. COPD Chronic Obstructive Pulmonary Disorders, early-stage COPD; a list of chemical compounds...) , what search engines were used (e.g. PubMed), what collection (MEDLINE, PubMed Central full-texts). Additionally quantitative details would be welcome also: how many abstracts or full-text articles were collected first?

I have read this submission. I believe that I have an appropriate level of expertise to confirm that it is of an acceptable scientific standard.

# Response

**Published:** 2015-05-12

**Authors:** Stephanie Boue

**Affiliations:** Philip Morris, Switzerland

Networks were built in multiple phases, as briefly described in M&M and in figure 1. The “original” networks were built in a non-disease context as described in detail in the respective publications [ 1-5]. Based on relevant reviews in the area and context of interest, research papers that report the causal mechanistic relationships relevant for a specific biological process were identified and the causal relationships were extracted and added to the model. Mechanistic relationships were also added from an existing Knowledgebase, a collection of causal relationships curated over more than ten years from over 40,000 papers and over 500,000 statements.

# Peer review: Referee response for version 1

**DOI:** 10.5256/f1000research.6402.r7526

**Published:** 2015-03-02

**Authors:** Gary Bader

**Affiliations:** 1 The Donnelly Centre, University of Toronto, Toronto, ON, Canada

High quality curation by trained database curators is needed in our community to convert the literature to computable models, but it is difficult to imagine how manual curation will scale to handle the ever-growing data generation rate in biology. Thus, the biological research community needs to figure out how to get crowdsourcing working for everyone as a tool to improve access to computable data. This paper does a very good job of describing how a set of COPD networks were constructed and enhanced (they grew in size and level of detail) through an interesting three-phase process. However, it would be useful to better describe the utility of the resulting networks and to further analyze the crowdsourcing process itself. Addressing these points will give the work a broader impact.

**Utility of networks:**

It is not clear what advantages the use of causal networks brings compared to more established models in the community, such as molecular interaction networks used by many algorithms (e.g. gene function prediction, module detection, interpretation of molecular profile data, network biomarkers) or detailed biochemical pathway models (used by most textbooks and pathway databases). While many results are published in terms of causal networks (e.g. A activates B), one important issue with networks constructed by collecting these relationships is that they may be difficult to integrate across resources since they are context specific: A may activate B in the lung, but inhibit B in the heart and when these are integrated, a conflict arises. Many computational analysis methods require integration of networks from multiple sources to construct the largest available network and integrate this data with disease-specific molecular profile data (e.g. gene expression data) to gain context (as it seems is done in the RCR approach). It would be useful for the authors to further discuss the utility of context-specific causal networks for follow on discovery.

I only noticed one sentence mentioning use: “By building the network model set in the BEL language format, we have generated a model framework suitable for biomarker discovery and for the interpretation of transcriptomic signatures found in human lung tissue.” However, this sentence is not clear and doesn’t cite any prior literature. How does using the BEL format create models suitable for biomarker discovery? Can’t molecular interaction or other types of networks also be used for biomarker discovery? What type of biomarker discovery is referred to here? How are transcriptomic signatures interpreted and analyzed?

**Crowdsourcing comments:**

“Networks that were not enhanced with COPD-specific mechanisms from the literature or RCR included the DNA Damage and Notch Signaling networks. Although both these networks relevant to the development of COPD, they were not augmented beyond the original, non-diseased network scaffolds, because no studies on the differences in signaling between non-diseased and diseased states were available.” How do the authors know that no relevant studies were available? It seems that many papers at least have discussed links between COPD and DNA damage or Notch signaling (e.g. PMID: 19106307 published in 2009 “Down-regulation of the notch pathway in human airway epithelium in association with smoking and chronic obstructive pulmonary disease.”)

“In total, 12 new nodes and 28 new edges were added to the Th1-Th2 Signaling network model during the jamboree discussions, thereby creating a comprehensive biological network of T-helper cell activity and their interactions with other immune cells in the context of COPD.”  How is ‘comprehensive’ measured? How do we know how much of the available literature was covered by the crowdsource process? That is, what is the sensitivity of the crowdsourcing process?

How many contributors were involved in enhancing each network in phase 2? Where were they from e.g. academia, industry? What incentivized them to contribute – for instance, were they COPD researchers? For the sake of research into crowdsourcing in biology, it would be very useful to provide additional analysis of the contributor community. We need to learn more about what works and what doesn’t in crowdsourcing initiatives so future generations of these approaches can be improved.

The authors state “With nearly 900 new pieces of evidence added by the challenge crowd, a significant overall enhancement of the networks was achieved in a relatively short time (5 months),” How many papers (PMIDs) supported the 900 pieces of evidence?

**Questions about use of BEL:**

Figure 4. The shorthand BEL notation is not widely recognized as a visual format and difficult to read in general. An easy to read visualization format would make the network figures much easier to understand. Also, what do the different edge end symbols (e.g. arrow, dot, diamond) mean?

Part C of Figure 1 mentions “BEL to openBEL conversion”. What’s the difference between BEL and openBEL?

**Other comments:**

A broader review of the literature of pathway databases and crowdsourcing efforts should be included in the introduction.

I have read this submission. I believe that I have an appropriate level of expertise to confirm that it is of an acceptable scientific standard.

# Response

**Published:** 2015-05-12

**Authors:** Stephanie Boue

**Affiliations:** Philip Morris, Switzerland

*High quality curation by trained database curators is needed in our community to convert the literature to computable models, but it is difficult to imagine how manual curation will scale to handle the ever-growing data generation rate in biology. Thus, the biological research community needs to figure out how to get crowdsourcing working for everyone as a tool to improve access to computable data. This paper does a very good job of describing how a set of COPD networks were constructed and enhanced (they grew in size and level of detail) through an interesting three-phase process. However, it would be useful to better describe the utility of the resulting networks and to further analyze the crowdsourcing process itself. Addressing these points will give the work a broader impact.*

**Authors’ response**: We have previously published several papers introducing use cases where the biological signal is interpreted in a meaningful manner using the causal network models [ref-1] ^-^[ref-7] and have added these references to support the statement in the text. The point of the reviewer is absolutely relevant and we acknowledge that it will be of utmost importance to critically assess how the usefulness of the networks changed through each phase of the network verification project. As a first step, the previously published analyses can be repeated with the crowd-verified networks to assess the impact of network verification on data interpretation. A thorough assessment of the impact of crowd verification, requires however an extensive analysis leveraging multiple relevant datasets and to be reported thoroughly would dissolve the intended content of this manuscript that concentrates on the way networks were built and later on verified and refined through a crowdsourcing approach. We will conduct such an analysis and include the reference as soon as it will become available. We have now addressed these points in the discussion.

***Utility of networks:***

*It is not clear what advantages the use of causal networks brings compared to more established models in the community, such as molecular interaction networks used by many algorithms (e.g. gene function prediction, module detection, interpretation of molecular profile data, network biomarkers) or detailed biochemical pathway models (used by most textbooks and pathway databases). While many results are published in terms of causal networks (e.g. A activates B), one important issue with networks constructed by collecting these relationships is that they may be difficult to integrate across resources since they are context specific: A may activate B in the lung, but inhibit B in the heart and when these are integrated, a conflict arises. Many computational analysis methods require integration of networks from multiple sources to construct the largest available network and integrate this data with disease-specific molecular profile data (e.g. gene expression data) to gain context (as it seems is done in the RCR approach). It would be useful for the authors to further discuss the utility of context-specific causal networks for follow on discovery.*

**Authors’ response:** The usage of causal networks allows all applications that other network models would have, and in addition eases the biological interpretation of the results in a mechanistic, cause and effect fashion. The new, sophisticated algorithms that have been developed to analyze molecular data using the causal network models fully exploit the specific structure of two-layer cause-and-effect network models, providing evidence that causality adds precision on top of interaction[ref-1] ^,^[ref-2] ^,^[ref-8]. However, as the reviewer points out, causality may differ across conditions (space and time), and the usage of BEL is therefore particularly relevant, as it allows for detailed context annotation of each piece of evidence linked to a causal edge. To fully make use of this property, it is important that as much of the literature evidence are collected in a knowledgebase, which will only really be doable thanks to new text mining methods assisting the biologists with the creation of BEL evidences or via crowdsourcing efforts such as the one described here. Because it is a very large undertaking, we have so far tried to restrict the evidences to respiratory and cardiovascular context. It is not excluded, however, that as the crowd and interest for the network grows, a more comprehensive annotation of the networks are achieved, making them usable in a specific context. Furthermore, BEL is being used in both academic and industry settings and BEL converters are being developed that can translate information from other sources such as BioPAX and SBML to facilitate comprehensive aggregation of networks.

*I only noticed one sentence mentioning use: “By building the network model set in the BEL language format, we have generated a model framework suitable for biomarker discovery and for the interpretation of transcriptomic signatures found in human lung tissue.” However, this sentence is not clear and doesn’t cite any prior literature. How does using the BEL format create models suitable for biomarker discovery? Can’t molecular interaction or other types of networks also be used for biomarker discovery? What type of biomarker discovery is referred to here? How are transcriptomic signatures interpreted and analyzed?*

**Authors’ response: **We have previously published several papers introducing use cases where the biological signal is interpreted in a meaningful manner using the causal network models[ref-1] ^-^[ref-7] and have added these references to support the statement in the text. Martin *et al. *describes the development of network signatures that identify mechanisms that may explain differential drug treatment response between individuals, demonstrating that the causal two layered networks allow analyses which go beyond what normal networks can provide, i.e. provide classification power coupled with mechanistic detail[ref-8].

***Crowdsourcing comments:***

*“Networks that were not enhanced with COPD-specific mechanisms from the literature or RCR included the DNA Damage and Notch Signaling networks. Although both these networks relevant to the development of COPD, they were not augmented beyond the original, non-diseased network scaffolds, because no studies on the differences in signaling between non-diseased and diseased states were available.” How do the authors know that no relevant studies were available? It seems that many papers at least have discussed links between COPD and DNA damage or Notch signaling (e.g. PMID: 19106307 published in 2009 “Down-regulation of the notch pathway in human airway epithelium in association with smoking and chronic obstructive pulmonary disease.”)*

**Authors’ response:** We reformulated the sentence. Although there may be papers that report on the correlation between COPD and these processes like the Notch paper you mention, we are referring to mechanistic papers that will provide causal links within the model. For example, a paper from a NOTCH1 knockout experiment in a COPD animal model that shows a particular protein being decreased will allow us to add the causal link of NOTCH1 activity increasing that protein in the Notch signaling COPD model. These are the types of causal mechanistic papers we have searched for and have not found in the context of COPD.

*“In total, 12 new nodes and 28 new edges were added to the Th1-Th2 Signaling network model during the jamboree discussions, thereby creating a comprehensive biological network of T-helper cell activity and their interactions with other immune cells in the context of COPD.”  How is ‘comprehensive’ measured? How do we know how much of the available literature was covered by the crowdsource process? That is, what is the sensitivity of the crowdsourcing process?*

**Authors’ response:** As to avoid any confusion, and because the sensitivity of crowdsourcing is not easily measurable (as it would require to assess all possible literature), we reformulated to “more comprehensive”.

*How many contributors were involved in enhancing each network in phase 2? Where were they from e.g. academia, industry? What incentivized them to contribute – for instance, were they COPD researchers? For the sake of research into crowdsourcing in biology, it would be very useful to provide additional analysis of the contributor community. We need to learn more about what works and what doesn’t in crowdsourcing initiatives so future generations of these approaches can be improved.*

**Authors’ response:** A specific publication addresses the statistics related to participation[ref-9]. Clearly, the most difficult part of such a crowdsourcing project is to get the right incentives for people to participate. We acknowledge that showing the usefulness of the networks and their refinements should allow for a bigger buy-in from the scientific community, and likely more participation.

*The authors state “With nearly 900 new pieces of evidence added by the challenge crowd, a significant overall enhancement of the networks was achieved in a relatively short time (5 months),” How many papers (PMIDs) supported the 900 pieces of evidence?*

**Authors’ response:** 479 unique PMIDs supported the 886 new pieces of evidence. We have included this detail in the text.

***Questions about use of BEL:***

*Figure 4. The shorthand BEL notation is not widely recognized as a visual format and difficult to read in general. An easy to read visualization format would make the network figures much easier to understand. Also, what do the different edge end symbols (e.g. arrow, dot, diamond) mean?*

**Authors’ response: **We have added a legend to the figure. Please note that the bionet website also has a legend for the network visualization part.

*Part C of Figure 1 mentions “BEL to openBEL conversion”. What’s the difference between BEL and openBEL?*

**Authors’ response:** BEL was a proprietary language developed by Selventa. In the interest of the growing community of researchers using BEL, an openBEL language derived from BEL has been developed and released as open source http://www.openbel.org/. One of the main differences between the two is that in the openBEL, the namespace (i.e. databases in which the biological entity is defined) is clearly stated, allowing for a better standardization of used ontologies and databases. We have added this specification in the figure legend.

***Other comments:***

*A broader review of the literature of pathway databases and crowdsourcing efforts should be included in the introduction.*

**Authors’ response:** We have discussed the comparison of our network models with other resources in other publications[ref-2] ^,^[ref-9] ^,^[ref-10]. We have added this statement with appropriate references in the discussion for readers, who wish to find more background information about the network models and see how they compared with other approaches to interpret data.

[References]

[[1|title=A network-based approach to quantifying the impact of biologically active substances|authors=Hoeng/J;Deehan/R;Pratt/D;Martin/F;Sewer/A;Thomson/TM;Drubin/DA;Waters/CA;de Graaf/D;Peitsch/MC|source=Drug discovery today|vol=17|issue=9-10|year=2012|fpage=413|lpage=418|type=journal|doi=10.1016/j.drudis.2011.11.008|pmid=22155224]]

[[2|title=Toxicopanomics: Applications of Genomics, Transcriptomics, Proteomics, and Lipidomics in Predictive Mechanistic Toxicology|authors=Hoeng/J;Talikka/M;Martin/F;Ansari/S;Drubin/D;Elamin/A;Gebel/S;Ivanov/NV;Deehan/R;Kogel/U;Mathis/C;Schlage/WK;Sewer/A;Sierro/N;Thomson/T;Peitsch/MC|source= In Hayes' Principles and Methods of Toxicology, Sixth Edition |vol= Edited by Hayes AW, Kruger CL|issue=CRC Press|year=2014|fpage=295|lpage=332| url=https://www.crcpress.com/product/isbn/9781842145364]]

[[3|title=A 28-day rat inhalation study with an integrated molecular toxicology endpoint demonstrates reduced exposure effects for a prototypic modified risk tobacco product compared with conventional cigarettes|authors=Kogel/U;Schlage/WK;Martin/F;Xiang/Y;Ansari/S;Leroy/P;Vanscheeuwijck/P;Gebel/S;Buettner/A;Wyss/C;Esposito/M;Hoeng/J;Peitsch/MC|source=Food and Chemical Toxicology|vol=38|year=2014|fpage=204|lpage=217|type=journal|doi=10.1016/j.fct.2014.02.034|pmid=24632068]]

[[4|title=A 7-month cigarette smoke inhalation study in C57BL/6 mice demonstrates reduced lung inflammation and emphysema following smoking cessation or aerosol exposure from a prototypic modified risk tobacco product|authors=Phillips/B;Veljkovic/E;Peck/MJ;Buettner/A;Elamin/A;Guedj/E;Vuillaume/G;Ivanov/NV;Martin/F;Boué/S|source=Food and Chemical Toxicology|vol=80|year=2015|fpage=328|lpage=345|type=journal|doi=10.1016/j.fct.2015.03.009|pmid=25843363]]

[[5|title=In vitro systems toxicology approach to investigate the effects of repeated cigarette smoke exposure on human buccal and gingival organotypic epithelial tissue cultures|authors=Schlage/WK;Iskandar/AR;Kostadinova/R;Xiang/Y;Sewer/A;Majeed/S;Kuehn/D;Frentzel/S;Talikka/M;Geertz/M|source=Toxicology mechanisms and methods|vol=24|issue=7|year=2014|fpage=470|lpage=487|type=journal|doi=10.3109/15376516.2014.943441|pmid=25046638|pmcid=PMC4219813]]

[[6|title=The Response of Human Nasal and Bronchial Organotypic Tissue Cultures to Repeated Whole Cigarette Smoke Exposure|authors=Talikka/M;Kostadinova/R;Xiang/Y;Mathis/C;Sewer/A;Majeed/S;Kuehn/D;Frentzel/S;Merg/C;Geertz/M|source=International Journal of Toxicology|vol=33|issue=6|year=2014|fpage=506|lpage=517|type=journal|doi=10.1177/1091581814551647|pmid=25297719]][[7|title= Quantitative assessment of biological impact using transcriptomic data and mechanistic network models|authors=Thomson/TM;Sewer/A;Martin/F;Belcastro/V;Frushour/BP;Gebel/S;Park/J;Schlage/WK;Talikka/M;Vasilyev/DM|source=Toxicology and Applied Pharmacology|vol=272|issue=3|year=2013|fpage=863|lpage=878|type=journal|doi=10.1016/j.taap.2013.07.007|pmid=23933166]]

[[8|title=: Quantification of biological network perturbations for mechanistic insight and diagnostics using two-layer causal models|authors=Martin/F;Sewer/A;Talikka/M;Xiang/Y;Hoeng/J;Peitsch/MC|source=BMC Bioinformatics|vol=15|year=2014|fpage=238|type=journal|doi=10.1186/1471-2105-15-238|pmid=25015298|pmcid=PMC4227138]]

[[9|title= Reputation-based collaborative network biology|authors=sbv IPT;Binder/J;Boue/S;Di Fabio/A;Fields/RB;Hayes/W;Hoeng/J;Park/JS;Peitsch/MC|source=Pacific Symposium on Biocomputing|year=2015|fpage=270|lpage=281|type=journal|doi=10.1142/9789814644730_0027 |pmid=25592588]]

[[10|title=Causal Biological Network (CBN) database: a comprehensive platform of causal biological network models focused on the pulmonary and vascular systems|authors=Boue/S;Talikka/M;Westra/JW;Hayes/W;Di Fabio/A;Park/J;Schlage/WK;Sewer/A;Fields/RB;Ansari/S;Martin/F;Veljkovic/E;Kenney/RD;Peitsch/MC;Hoeng/J|source= Database|vol=2015|issue=bav030 |year=2015|fpage=1|lpage=14|type=journal|doi=10.1093/database/bav030|pmid=25887162]]

# Peer review: Referee response for version 1

**DOI:** 10.5256/f1000research.6402.r7528

**Published:** 2015-02-06

**Authors:** Winston Hide

**Affiliations:** 1 Department of Biostatistics, Harvard School of Public Health, Boston, MA, USA

This work uses a hybrid approach network modelling approach to incorporate predictive methodology with empirical knowledge and crowd sourcing for models of COPD pathogenesis. It is a good idea, thoroughly implemented and has produced a potentially useful set of pathways. The value of the resulting pathways is not clear as they do not have community validation, only community design. The manuscript is exhaustive in its descriptions and the process of developing the models is clear.

The work represents the first phase of understanding for knowledge driven development of network models of COPD - the process of building the models is well described and the actual outcomes of the interactions with community are informative. The question of the actual true value of the models in terms of their accuracy, adoption and  accessibility is not yet convincingly addressed. That may be expected as the purpose of this work appears to be a description of the first part of the process of developing knowledge based models for a disease. The models as presented appear unvalidated and without a description of the framework for assessing the value and actioning of the networks, it is not clear how their uptake by the community will be assured.

This is a unique effort but the manuscript should make more reference to existing pathway based community annotation efforts e.g.: wikipathways and/or open science initiatives such as those promoted by community interaction leaders such as Andrew Su. It should show how the value of this approach differs to existing efforts.

In terms of access to expertise, it is not clear how an uninvited scientist would contribute to an existing pathway model - except through the open but time-limited crowdsourcing venue.

Straightforward validation of the models network is not tested in terms of their consistency or cross-valdiation within COPD high dimensional assays - where it should be possible to see evidence of enrichment for co-expression etc.

Contextual nature of networks is mentioned and attempts are made to address contextual pathway structures, but the context is not tested.

As a suggestion the authors should consider community validation

Pathway accessibility and distribution is described but it is not clear as to how these models are available in any format except web browsing. For the models to be tested by the community, value would come from making them openly available as downloadable instances in several of the most popular formats. Feedback on their accuracy could then be encouraged.

I have read this submission. I believe that I have an appropriate level of expertise to confirm that it is of an acceptable scientific standard.

# Response

**Published:** 2015-05-12

**Authors:** Stephanie Boue

**Affiliations:** Philip Morris, Switzerland

*This work uses a hybrid approach network modelling approach to incorporate predictive methodology with empirical knowledge and crowd sourcing for models of COPD pathogenesis. It is a good idea, thoroughly implemented and has produced a potentially useful set of pathways. The value of the resulting pathways is not clear as they do not have community validation, only community design. The manuscript is exhaustive in its descriptions and the process of developing the models is clear.*

*The work represents the first phase of understanding for knowledge driven development of network models of COPD - the process of building the models is well described and the actual outcomes of the interactions with community are informative. The question of the actual true value of the models in terms of their accuracy, adoption and  accessibility is not yet convincingly addressed. That may be expected as the purpose of this work appears to be a description of the first part of the process of developing knowledge based models for a disease. The models as presented appear unvalidated and without a description of the framework for assessing the value and actioning of the networks, it is not clear how their uptake by the community will be assured.*

**Authors’ response:** The point of the reviewer is absolutely relevant and we acknowledge that it will be of utmost importance to critically assess how the usefulness of the networks changed through each phase of the project. Whenever possible, orthogonal data sets were used to validate the network model during the building process. In the paper *Systematic verification of upstream regulators of a computable cellular proliferation network model on non-diseased lung cells using a dedicated dataset*, we have done just that by using a specifically designed, independent lung cell proliferation dataset to verify the correctness of the cell cycle network model [ref-1]. The validation of all available networks requires an extensive analysis leveraging multiple relevant datasets and to be reported thoroughly would dissolve the intended content of this manuscript that concentrates on the way networks were built and later on verified and refined through a crowdsourcing approach. We will conduct such an analysis and make sure to reference it here as soon as it will be available.

*This is a unique effort but the manuscript should make more reference to existing pathway based community annotation efforts e.g.: wikipathways and/or open science initiatives such as those promoted by community interaction leaders such as Andrew Su. It should show how the value of this approach differs to existing efforts.*

**Authors’ response: **We have discussed the comparison of our network models with other resources in other articles[ref-2] ^,^[ref-3]  and in a book chapter[ref-4]. We have added this statement in the discussion for readers who wish to find more background information about the network models and see how they compared with other approaches to interpret data.

*In terms of access to expertise, it is not clear how an uninvited scientist would contribute to an existing pathway model - except through the open but time-limited crowdsourcing venue.*

Straightforward validation of the models network is not tested in terms of their consistency or cross-valdiation within COPD high dimensional assays - where it should be possible to see evidence of enrichment for co-expression etc.

*Contextual nature of networks is mentioned and attempts are made to address contextual pathway structures, but the context is not tested.*

*As a suggestion the authors should consider community validation*

*Pathway accessibility and distribution is described but it is not clear as to how these models are available in any format except web browsing. For the models to be tested by the community, value would come from making them openly available as downloadable instances in several of the most popular formats. Feedback on their accuracy could then be encouraged.*

**Authors’ response**: The networks can be browsed on the bionet.sbvimprover.com website, including latest votes and modification. More stable versions are stored in the causalbionet.com database[ref-2].

[References]

[[1|title=Systematic verification of upstream regulators of a computable cellular proliferation network model on non-diseased lung cells using a dedicated dataset. Bioinformatics and biology insights|authors=Belcastro/V;Poussin/C;Gebel/S;Mathis/C;Schlage/WK;Lichtner/RB;Quadt-Humme/S;Wagner/S;Hoeng/J;Peitsch/MC|source=Bioinformatics and biology insights|vol=7|year=2013|fpage=217|lpage=230|type=journal|doi=10.4137/BBI.S12167|pmid=23926424|pmcid=PMC3733638]]

[[2|title=Causal Biological Network (CBN) database: a comprehensive platform of causal biological network models focused on the pulmonary and vascular systems|authors=Boue/S;Talikka/M;Westra/JW;Hayes/W;Di Fabio/A;Park/J;Schlage/WK;Sewer/A;Fields/RB;Ansari/S;Martin/F;Veljkovic/E;Kenney/RD;Peitsch/MC;Hoeng/J|source= Database|vol=2015|issue=bav030 |year=2015|fpage=1|lpage=14|type=journal|doi=10.1093/database/bav030|pmid=25887162]]

[[3|title= Reputation-based collaborative network biology|authors=sbv IPT;Binder/J;Boue/S;Di Fabio/A;Fields/RB;Hayes/W;Hoeng/J;Park/JS;Peitsch/MC|source=Pacific Symposium on Biocomputing|year=2015|fpage=270|lpage=281|type=journal|doi=10.1142/9789814644730_0027 |pmid=25592588]]

[[4|title=Toxicopanomics: Applications of Genomics, Transcriptomics, Proteomics, and Lipidomics in Predictive Mechanistic Toxicology|authors=Hoeng/J;Talikka/M;Martin/F;Ansari/S;Drubin/D;Elamin/A;Gebel/S;Ivanov/NV;Deehan/R;Kogel/U;Mathis/C;Schlage/WK;Sewer/A;Sierro/N;Thomson/T;Peitsch/MC|source= In Hayes' Principles and Methods of Toxicology, Sixth Edition |vol= Edited by Hayes AW, Kruger CL|issue=CRC Press|year=2014|fpage=295|lpage=332| url=https://www.crcpress.com/product/isbn/9781842145364]]
